# Reducing Overutilization of High-flow Nasal Cannula in Children with Bronchiolitis

**DOI:** 10.1097/pq9.0000000000000690

**Published:** 2023-10-07

**Authors:** Diana Jo, Nisha Gupta, David Bastawrous, Hayley Busch, Asha Neptune, Amy Weis, Courtney Port

**Affiliations:** From the * Department of Pediatrics, Inova Children’s Hospital, Falls Church, Va.; † Division of Pediatric Hospital Medicine, Inova Children’s Hospital, Falls Church, Va.

## Abstract

**Background:**

Bronchiolitis is a leading cause of pediatric hospitalizations. A high-flow nasal cannula (HFNC) does not significantly improve clinical outcomes and is associated with increased costs and intensive care unit (ICU) utilization. Despite this, hospitals continue to overuse HFNC in children with bronchiolitis. We aimed to reduce HFNC initiation in children hospitalized with bronchiolitis by 20 percentage points within 6 months.

**Methods:**

This study included patients aged 1 month to 2 years diagnosed with bronchiolitis, excluding patients with prematurity less than 32 weeks or preexisting cardiopulmonary, genetic, congenital, or neuromuscular abnormalities. Measures included HFNC utilization, length of stay, length of oxygen supplementation (LOOS), ICU transfers, and emergency department (ED) revisits and readmissions. For our primary intervention, we implemented a HFNC initiation protocol incorporating a respiratory scoring system, a multidisciplinary care-team huddle, and an emphasis on supportive care. Staff education, electronic health record integration, and audit and feedback were used to support implementation. Statistical process control charts were used to track metrics.

**Results:**

We analyzed 325 hospitalizations (126 baseline and 199 postintervention). The proportion of children hospitalized with bronchiolitis who received HFNC decreased from a mean of 82% to 60% within 1 month of implementation. Length of stay decreased from a median of 54 to 42 hours, and length of oxygen supplementation decreased from 50 to 38 hours. There were no significant changes in ICU transfers, 7-day ED revisits, or readmissions.

**Conclusions:**

Implementing a HFNC initiation protocol can safely reduce the overutilization of HFNC in children hospitalized with bronchiolitis.

## INTRODUCTION

Viral bronchiolitis is a leading cause of emergency department (ED) visits and hospitalizations in children younger than two years old.^1,2^ High-flow nasal cannula (HFNC) has become increasingly used for children hospitalized with bronchiolitis despite the lack of significant improvements in clinical outcomes.^3,4^ Studies show that HFNC does not significantly improve the length of stay (LOS) or length of oxygen supplementation (LOOS) but can be associated with increased healthcare costs and intensive care unit (ICU) utilization.^3–6^ Recent studies have demonstrated that HFNC can be used as rescue therapy for patients in severe respiratory distress when standard oxygen therapy fails.^7,8^ Given HFNC’s limited clinical benefits, associated costs, and the self-limited nature of the illness,^9^ it is imperative to reduce the overutilization of HFNC in children hospitalized with bronchiolitis.

Previous studies have successfully reduced the use of HFNC using quality improvement (QI) methodology.^10–12^ These studies used weaning protocols to decrease the total duration of HFNC supplementation or LFNC trials to reduce the initiation of HFNC. Although such protocols are important, they lack guidance on when to initiate HFNC. In a recent commentary, Treasure et al calls for providers to start aggressively deimplementing HFNC “through scholarly QI, with the next step hopefully addressing which patients to start on this costly, often misunderstood therapy.”^13^

Our institution experienced therapeutic creep, whereby HFNC became standardized therapy for most children hospitalized with bronchiolitis. Therefore, we sought to reduce overutilization and hypothesized that implementing a HFNC initiation protocol would aid deimplementation efforts. We aimed to decrease the proportion of children hospitalized with bronchiolitis who received HFNC from a mean of 82% to 62% within 6 months. We felt a 20 percentage point decrease in utilization was appropriate for a treatment modality entrenched in the local culture and comparable to reductions achieved by a similar study.^12^

## METHODS

### Context

This project was a single-center QI study at a 226-bed, Mid-Atlantic academic children’s hospital. An average of 45,000 pediatric ED visits occur, with approximately 7000 children admitted to our general pediatric unit each year. At the beginning of this project, HFNC flow rates at our institution were grouped by weight. Initiation of HFNC was recommended as low as 0.5 L/kg depending on weight category. Maximum rates for the floors were approximately 1 L/kg, and for the intermediate care unit (IMC) were 2 L/kg. Patients transferred to the IMC continue on the pediatric hospitalist service, and patients requiring >2 L/kg were generally transferred to the ICU. There was no standard for initiating low-flow nasal cannula (LFNC) in relation to HFNC utilization.

Project team members included pediatric resident physicians, pediatric hospitalists, pediatric ED physicians, pediatric intensivists, a QI expert, a clinical nurse director, a clinical nurse educator, and the director of respiratory therapy (RT) services. Stakeholders met monthly before the intervention period to develop the processes and metrics.

### Population

We included hospitalized patients between 1 month and 2 years of age admitted to the general pediatric unit diagnosed with bronchiolitis. We excluded patients born at less than 32 weeks gestation or with pre-existing cardiopulmonary conditions or genetic, congenital, or neuromuscular abnormalities. Five authors (DJ, NG, DB, HB, and CP) conducted data extraction. They ensured patients were within the inclusion and exclusion criteria by exploring the patient’s problem list and physician documentation within the history and physical. Data extractors met to review problems and reach a consensus during baseline data collection. We also excluded patients for whom the ICU or another facility initiated oxygen therapy before arriving at our general pediatric unit. We included patients for which our pediatric ED initiated oxygen therapy. We reviewed the charts of discharged patients with an encounter diagnosis of bronchiolitis for inclusion and exclusion criteria.

### Understanding the Problem

**Fig. 1.**
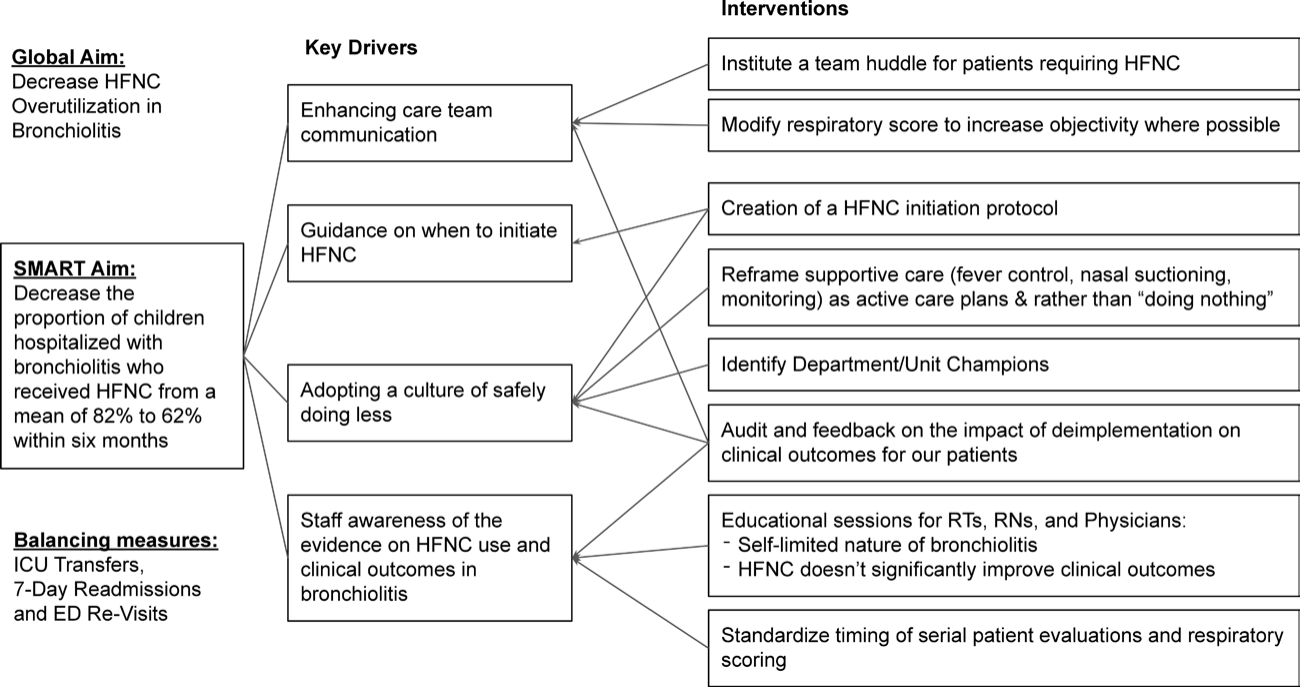
Key driver diagram.

Project stakeholders reviewed existing literature and developed a unified understanding of the project goals and key contributors to success by creating a key driver diagram (Fig. 1). A prior study at this organization studied providers’ acceptability of HFNC deimplementation amongst various roles and clinical settings. Results showed that a majority of providers found the deimplementation of HFNC acceptable.^14^ We used barriers and facilitators identified from this study to aid in developing key drivers and targeted interventions. This study grouped barriers to deimplementation into themes: discomfort with not intervening, the perception that HFNC has nonevidence-based clinical benefit, and variation in risk tolerance and clinical experience. Facilitators promoting deimplementation included education for staff, adopting a culture of safely doing less, and enhanced multidisciplinary communication.

We theorized that individual provider efforts could not overcome high rates of HFNC initiation at our institution but that an institutional change was necessary. We used The Model for Improvement framework to test interventions for meaningful outcome change.^15^

### Intervention

To promote a culture of safely doing less, we developed and implemented a HFNC initiation protocol (**Supplementary Appendix A**, http://links.lww.com/PQ9/A517) that emphasized initial supportive care via fever control and nasal suctioning, then waiting 30 minutes to obtain a respiratory score. We modified our institutional respiratory score from a numerical to a categorical score based on inter-departmental discussions and a review of other hospitals’ scoring systems. We scored patients as mild, moderate, or severe based on the highest rating in any category (respiratory rate, work of breathing, mental status, and breath sounds). Patients scoring mild to moderate were scored again within 30 to 60 minutes. If the score remained mild or moderate, the patient would either be discharged home or admitted to the pediatric hospitalist service for observation without initiating HFNC and scored every two to four hours and as needed for worsening clinical status. Patients with a severe respiratory score or those who were unable to maintain oxygen saturations 90% or more on LFNC prompted the initiation of a multidisciplinary bedside huddle with the patient’s physician (resident physician, advanced practice provider, or an attending), RT and nurse (RN) to develop consensus on the assessment and management plan. Patients were then either re-evaluated within an hour or initiated on HFNC.

### Implementation Strategies

We held live and recorded department-based staff education sessions. During these sessions, we reviewed the evidence of HFNC in children with bronchiolitis and physical exam findings seen in children with respiratory distress (eg, retractions, head bobbing, and grunting) via a short video.^16^ We also discussed supportive therapies as alternative treatment options and communication strategies when encountering care-team members who felt uncomfortable with the patient’s clinical status. Lastly, we introduced the HFNC initiation protocol, and providers participated in case-based scenarios to navigate the protocol.

We used several strategies to improve the adoption and implementation of the HFNC initiation protocol. First, we integrated a note template to document a patient’s respiratory score and care plan into the electronic health record (EHR). This template included drop-down lists for quick scoring and a built-in clinical decision tool offering intervention options aligned with the HFNC initiation protocol. QI team members performed chart reviews in near real-time to ensure that providers correctly used the note template during the first weeks of implementation and provided individual feedback whenever possible. Secondly, we performed an audit and feedback to address concerns that HFNC deimplementation may result in adverse clinical outcomes, and then we distributed results during departmental meetings and via email.

### Evolution of Interventions Over Time

The HFNC initiation protocol went through multiple revisions based on stakeholder feedback. We added a section for initiating HFNC within the ED, including guidance on monitoring time and disposition. Since we reserved HFNC use for severe symptoms and as a rescue therapy, we suggested starting HFNC flow rates at 1–1.5 L/kg. We subsequently adjusted the maximum flow rates for general pediatric and intermediate care units to approximately 1.5 and 2 L/kg, respectively.

In response to poor documentation of respiratory scores and scores not reflecting the highest score in any category, authors provided one-on-one coaching to RTs during 2 consecutive day-time shifts. They gave feedback at a weekly RT departmental meeting. Feedback obtained from RTs during these sessions led to the optimization of the note template, including adding a comment that the single highest rating in any one category dictates the patient’s respiratory score. We also added other frequently used treatment interventions to the drop-down list.

### Measures

The primary outcome measure was the proportion of children hospitalized with bronchiolitis initiated on HFNC. Additional outcome measures included the proportion of children treated with LFNC or observed without supplemental oxygen therapy, LOS, and LOOS. We calculated LOS based on the admission and discharge times listed within the encounter. We calculated LOOS based on the length of time from the first documentation of oxygen supplementation to the first documentation of room air within the vital signs EHR flow sheet. When a child was placed back on oxygen therapy after weaning to room air, the LOOS included the time on room air between periods of oxygen supplementation.

Process measures included the proportion of children with a documented respiratory score and a severe respiratory score at the time of HFNC initiation. In addition, we created a conversion to equate the previous numerical and the new categorical respiratory scores (0–2 = mild, 3–5 = moderate, 6-7 = severe). Balancing measures included the proportion of children transferred to the ICU and those with ED revisits or readmissions for respiratory concerns within 7 days of hospital discharge. We limited ED revisits and readmissions to those encounters available within our EHR system.

### Data Collection

We collected baseline data retrospectively from November 2019 to March 2021. It is important to note that we had extremely low numbers during the 2020–2021 bronchiolitis season due to the impact of the COVID-19 pandemic on overall pediatric infections and hospitalizations, including bronchiolitis.^17–19^ Authors collected postintervention data every two weeks from October 2021 to April 2022. We extracted data from our EHR (Epic Systems, Verona, Wis.) and stored it in REDCap (Vanderbilt University, Nashville, Tenn.), a Health Insurance Portability and Accountability Act-compliant software.

### Analysis

We used descriptive statistics to compare baseline and postintervention groups. We used chi-square and Fisher exact tests to compare patient characteristics, the proportion of children requiring escalation to the ICU, and the proportion of children with 7-day ED revisits or readmissions. A *P* value of ≤ 0.05 was considered statistically significant.

We analyzed children with documented respiratory and severe respiratory scores at the initiation of HFNC via separate run charts developed using QI Macros in Microsoft Excel. Median lines were adjusted based on accepted rules: shifts of six or more consecutive data points above or below the median and trends of five consecutive data points in increasing or decreasing order. We created statistical process control charts for subgroups of children treated with LFNC, HFNC, or both LFNC and HFNC. We made centerline adjustments when eight consecutive data points were above or below the mean. We defined special-cause variation using the Montgomery rules.^20^

We conducted a subanalysis for LOS and LOOS, which excluded the 2020–2021 bronchiolitis season. Given the COVID-19 pandemic significantly impacted this season and was not representative of our usual clinical practice, the 2019–2020 respiratory season represented a more accurate baseline for LOS and LOOS. We also used descriptive statistics to compare baseline and postintervention groups for LOS and LOOS using Mann-Whitney U tests.

### Ethical Considerations

This project was a QI study exempted from our local institutional review board.

## RESULTS

This study analyzed 325 hospitalized children with bronchiolitis, 126 from baseline and 199 from postintervention. Table 1 compares baseline and postintervention patient characteristics. During the baseline period, children were younger, less likely to be premature, more likely to have tested positive for the respiratory syncytial virus, and had a symptom duration greater than 2 days.

**Table 1.** Baseline and Postintervention Patient Characteristics

	Baseline(*n *= 126)		Postintervention(*n *= 199)		*P*
	#	%	#	%	
<3 months old	87	69%	45	23%	<0.001^*^
3–12 months old	15	12%	87	44%	<0.001^*^
1–2 years	24	19%	67	34%	0.004^*^
≥37 weeks gestation	110	87%	156	78%	0.042^*^
≤36 weeks gestation	12	10%	24	12%	0.48^*^
Gestational age not available	4	3%	19	10%	0.04^†^
Women	40	32%	67	34%	0.72^*^
Symptom duration ≥5 days	35	28%	53	27%	0.82^*^
Symptom duration 3–4 d	61	48%	81	41%	0.17^*^
Symptom duration 2 d or less	16	13%	62	31%	<0.001^*^
Unknown duration	14	11%	3	2%	<0.001^†^
Respiratory syncytial virus positive	82	65%	91	46%	<0.001^*^

^*^X^2^ testing.

^†^Fisher exact test.

**Fig. 2.**
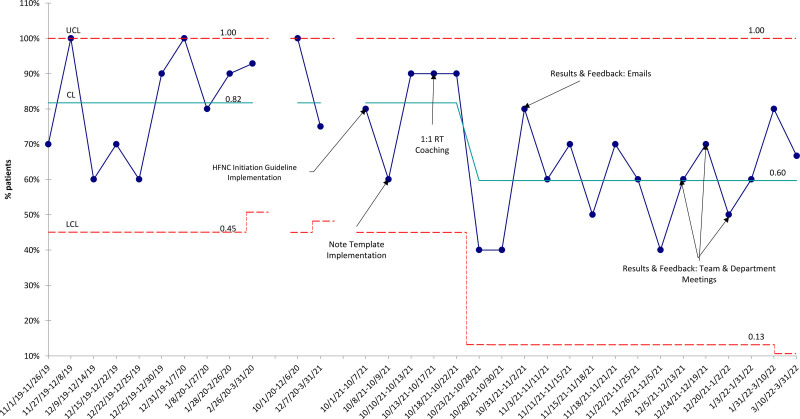
High-flow nasal cannula utilization in children hospitalized with bronchiolitis. Statistical process control *P* chart demonstrating the proportion of children hospitalized with bronchiolitis who received HFNC treatment over time. LCL = lower control limit; CL = centerline; UCL = upper control limit.

**Fig. 3.**
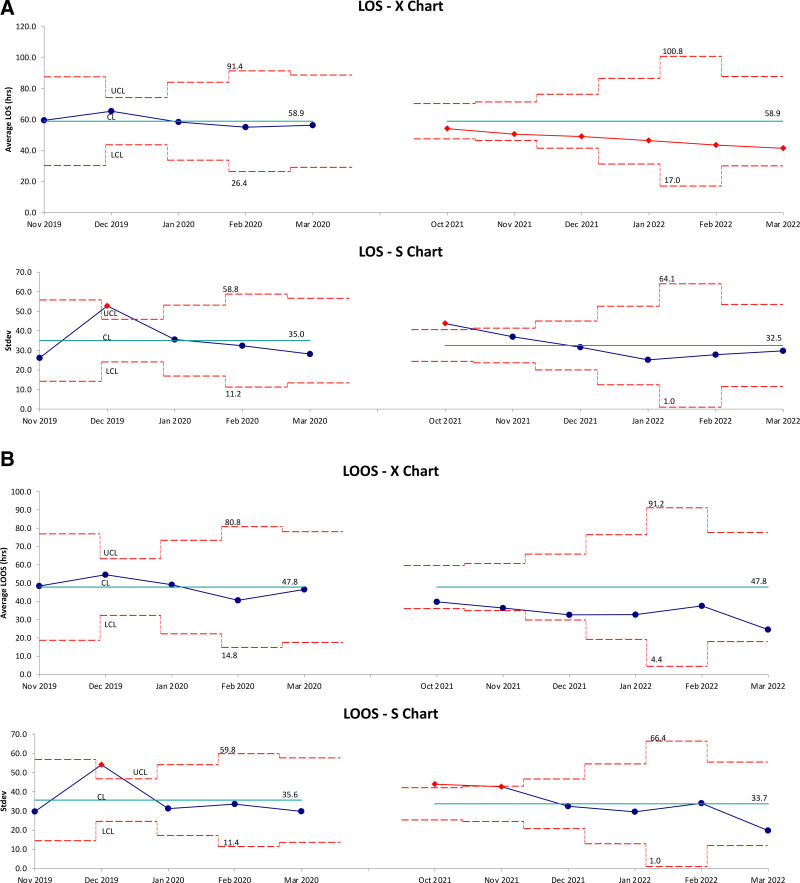
Length of stay and length of oxygen supplementation over time. Statistical process control X and S charts representing LOS (A) and LOOS (B) over time. LCL = lower control limit; CL = centerline; UCL = upper control limit.

The proportion of children hospitalized with bronchiolitis who received HFNC decreased from a mean of 82%–60% (Fig. 2). LOS and LOOS for the 2020–2021 season were unusually low and lower than our postintervention improvements (medians of 30 hours and 26 hours, respectively). When analyzing LOS and LOOS for the 2019–2020 and 2021–2022 seasons, there was a trend toward improvement, but the centerline did not shift (Fig. 3A and B).

**Fig. 4.**
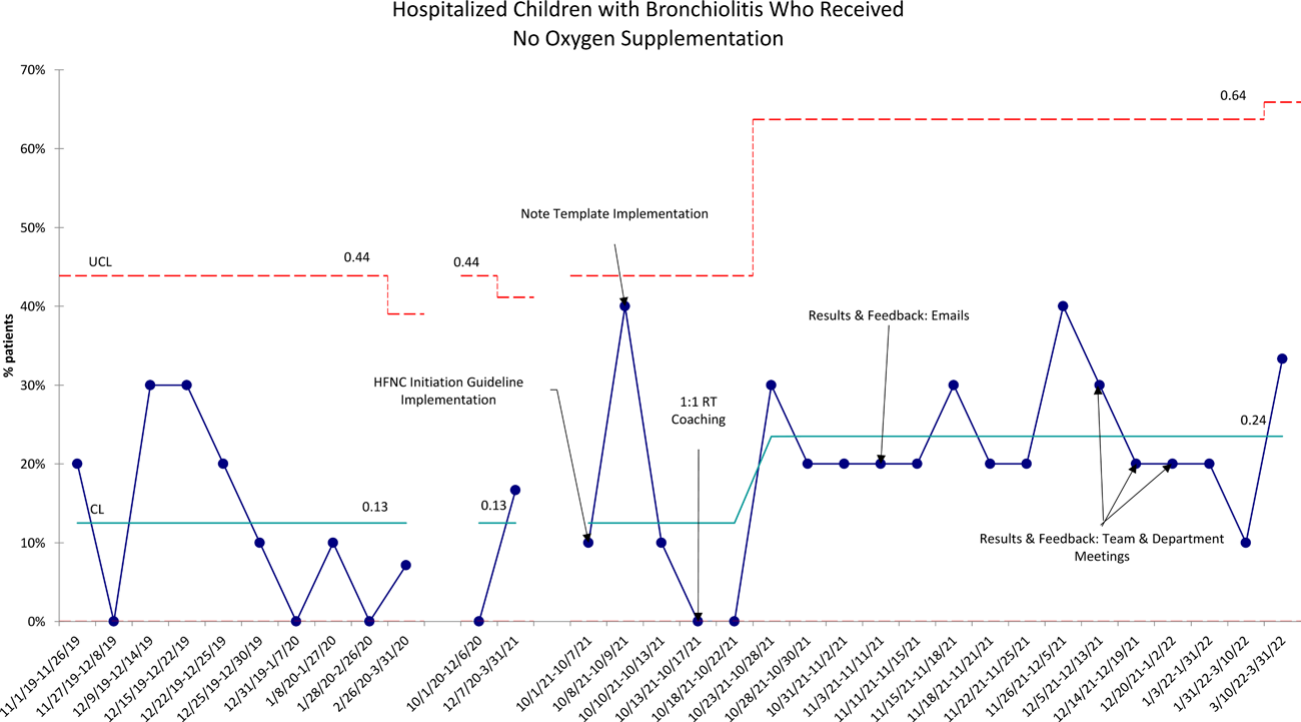
Hospitalized children with bronchiolitis who received no oxygen supplementation. Statistical process control *P* chart representing hospitalized children with bronchiolitis who received no oxygen supplementation. Each data point contains 10 consecutive patients, except for the final baseline (n = 14) and the final postintervention (n = 9) data points. CL = centerline; UCL = upper control limit.

Although not the target of our interventions, there were also changes in the utilization of LFNC and the proportion of children observed without oxygen therapy. LFNC use increased from a mean of 18%–41% and then decreased to 26% for the remainder of the study. The proportion of children receiving both HFNC and LFNC supplemental oxygen remained the same at a mean of 14%. Finally, there was an increase in the proportion of children over time who did not receive any supplemental oxygen during their hospital stay, from a mean of 13%–24% (Fig. 4).

The proportion of children with a documented respiratory score at HFNC initiation decreased during the baseline period but returned to a similar mean. The proportion of children with a severe respiratory score at HFNC initiation increased from a mean of 0%–20%.

There were no significant differences in the proportion of children requiring escalation to the ICU (baseline 1.6% (n = 2) and postintervention 2.5% (n = 5), *P = 0.71*); 7-day ED revisits (baseline 3.2% (n = 4) and postintervention 1.7% (n = 2), *P = 0*.21); or 7-day readmissions (baseline 1.6% (n = 2) and postintervention 1.7% (n = 2), *P = 0*.64).

## DISCUSSION

We achieved our aim of reducing HFNC utilization for children hospitalized with bronchiolitis using a HFNC initiation protocol. Additionally, we saw a decrease in LOS and LOOS when comparing the 2019–2020 and 2021–2022 seasons without negatively impacting rates of ICU transfers, 7-day ED revisits, or readmissions. Our main intervention was a multifaceted tool that guided when to initiate HFNC and incorporated alternative supportive therapies and a multidisciplinary team huddle.

HFNC protocols have been shown to decrease the duration of HFNC utilization and LOS.^10–12^ Charvat et al decreased the duration of HFNC treatment by 17 hours and LOS by 23 hours via the implementation of a rapid weaning protocol; however, they found an increase in the number of patients who were initiated on HFNC during their intervention period.^10^ They concluded that some of their success in decreasing LOS might lie in weaning patients who never needed HFNC. Noelck et al decreased the duration of HFNC for patients on 8 L or less per minute of HFNC, an a priori-determined waste line, from 36 to 18 hours and LOS from 4.1 to 3 days via a standard trial off of HFNC for eligible patients.^11^ However, Noelck et al did not find a significant change in the mean total hours of HFNC. Although these studies successfully decreased LOS and/or duration of HFNC utilization, they do not guide how to decrease HFNC initiation. Our study adds to the literature by demonstrating the success of using a HFNC initiation protocol to reduce HFNC initiation, LOS, and LOOS.

Previous studies provide limited information on which patients would benefit from HFNC.^21^ Our HFNC initiation protocol guides providers on when to initiate HFNC and builds on previously described efforts. Siraj et al reduced HFNC initiation from 62% to 43% by implementing a LFNC trial before HFNC initiation.^12^ Although our protocol did not recommend starting LFNC as a proxy, we nevertheless found an increase in LFNC utilization from 18% to 41%, subsequently dropping to 26%. This increase may have resulted from providers utilizing LFNC as an alternative to HFNC. One noteworthy finding was our high baseline of HFNC utilization. This high baseline is likely the result of differences in institutional practice and culture surrounding HFNC utilization, as demonstrated by a prior institutional study on understanding the acceptability of HFNC deimplementation.^14^ Providers described discomfort with not intervening and the perception that HFNC helps patients appear more comfortable. We hypothesized that the utilization of HFNC at our institution had become synonymous with hospitalization for bronchiolitis, resulting in a cycle of increasing utilization. Therefore, future studies should identify risk factors for children at a higher risk for acute respiratory decompensation.

Decreasing healthcare costs associated with bronchiolitis admissions was not an aim of this study, yet decreasing HFNC utilization may also be a cost-saving measure. Cost savings are frequently tied directly to LOS, and our study safely reduced LOS. Although healthcare delivery costs vary between healthcare systems, there is potential for healthcare savings by decreasing HFNC utilization, particularly in hospitals where HFNC can only be managed in the ICU.

Many factors likely contributed to our project’s success. We targeted primary drivers by gathering a team of stakeholders from different departments and provider roles and identified unit-based project champions. Creating a shared mental model was imperative for stakeholders’ buy-in, optimizing feedback, and promoting institutional culture change. The aforementioned qualitative study completed at our institution allowed for identifying local barriers and facilitators to HFNC deimplementation and developing subsequent targeted interventions. Additionally, surveying all providers about their thoughts surrounding HFNC deimplementation, including how they feel regarding deimplementation, how much effort it will take, how well it aligns with their values, and how difficult it will be. This survey may have instilled a sense of being heard and valued and eased some of the inevitable resistance encountered when implementing change.

Our QI study is limited in its generalizability because it was a single-center study with a high baseline rate of HFNC utilization. Despite this, institutions with lower baseline HFNC utilization can still use this study to standardize HFNC initiation. Further studies will be needed to see if such practices result in similar reductions in HFNC overutilization. In addition, we found that patients from the baseline group differed from those in the postintervention group in potentially meaningful ways. First, as a younger cohort with a longer duration of symptoms, the baseline group may have been more likely to develop severe symptoms necessitating HFNC therapy. Secondly, our previous numerical respiratory score did not easily convert into a categorical score, limiting comparisons of respiratory scores at HFNC initiation over time. This finding is evident because no patients scored in the severe category during the baseline study period. Although we successfully achieved our aim, only 20% of patients initiated on HFNC had documented severe respiratory scores. Although both respiratory and severe respiratory score documentation at HFNC initiation show increasing trends, we need additional data to see if further improvements are achieved and sustained. Lastly, we observed an initial increase in LFNC utilization and a subsequent decline. No changes were made to the guideline to suggest a trial of LFNC instead of HFNC. However, providers may have used LFNC instead of HFNC if patients did not meet the criteria for a severe respiratory score. The next steps include efforts to further reduce the overutilization of HFNC, given our still high postintervention rates based on the literature.^12,22^ We intend to incorporate a trial off of HFNC for incoming transported patients arriving with a mild to moderate respiratory score while on HFNC.

## CONCLUSIONS

Our QI study safely reduced the overutilization of HFNC, LOS, and LOOS for children hospitalized with bronchiolitis by implementing a HFNC initiation protocol. Healthcare providers can adapt and implement this protocol at their local institutions to improve the quality of care for children hospitalized with bronchiolitis. Future studies should consider implementing a protocol incorporating HFNC initiation guidance and rapid weaning.

## DISCLOSURE

The authors have no financial interest to declare in relation to the content of this article.

## Supplementary Material
