# Supplementary material for: Reducing Overutilization of High-flow Nasal Cannula in Children with Bronchiolitis
Source: Pediatr Qual Saf. 2023 Oct 7;8(5):e690. doi: 10.1097/pq9.0000000000000690 (PMC10561806; doi:10.1097/pq9.0000000000000690)
Supplement: Supplementary file 1 [file pqs-8-e690-s001.pdf]

Supplementary Appendix A: Pediatric high flow nasal cannula initiation protocol

Pediatric Humidified High Flow Nasal Cannula Initiation Guideline (HFNC)

Updated: 8/12/21 N.G.

<u>Supportive Care</u>
Suction and treat fever
Notify MD if O2 sat ≤90%, consider LFNC
Check O2 sat q4h unless on oxygen

↓

Obtain respiratory score (RS) 30 minutes after performing above

If RS **MILD OR MODERATE**

↓

<u>ED</u> – re-score within 30-60 minutes, if RS remains mild/moderate → dispo home or admit for obs

<u>Gen Peds</u> - supportive care (see above)
RT/MD re-score q2-4h and PRN

If RS **SEVERE** <u>OR</u>
Unable to keep O2 ≥ 90% on LFNC

↓

<u>HUDDLE</u> with MD, RT and RN and determine if HFNC appropriate. RT document score and huddle decision

If HFNC to be initiated see guidelines below

If HFNC not initiated, RT/MD to re-score within 30-60 minutes, if RS remains severe, consider HFNC

Respiratory Score (RS)

		Mild	Moderate	Severe
Respiratory Rate	< 3 mo	30-60	61-80	> 80
	3 - 11 mo	25-50	51-70	> 70
	1y – 3y	20-40	41-60	> 60
	≥ 4 y	12-20	21-30	> 30
Work of Breathing		Subcostal or intercostal	**2 of the** following: (subcostal, intercostal, suprasternal, nasal flaring)	**3 or more** (subcostal, intercostal, suprasternal, nasal flaring) **OR** head bobbing **OR** grunting
Mental Status		Playful but less active than usual	Fussy but consolable, tired appearing	Lethargic, inconsolable, or cyanotic
Breath Sounds		Good aeration	Fair aeration	Poor aeration

The single highest rating in any category dictates the patient's current assessment

<u>HHFNC Flow Initiation Guide</u>

Initiate flow at 1-1.5L/kg @ 40% FiO2

<u>ED initiation</u> - re-score within 30-60 min
If RS improved and ≤ max flow for IMC → transfer
if RS not improved → increase flow as directed and consider PICU if needed

<u>Gen Peds initiation</u> - re-score within 60 min
If RS improved → follow weaning protocol
If RS not improved → increase flow as directed, re-score within 30-60 min, consider PICU if needed

Patient Weight	Increase flow by	Max Flow for GEN PEDS (Max FiO2 60%)	Max flow for IMC STATUS
<10 kg	2 LPM	10 LPM	15 LPM
10-15 kg	2 LPM	15 LPM	20 LPM
15-20 kg	5 LPM	20 LPM	25 LPM
20-40kg	5 LPM	25 LPM	30 LPM
>40kg	5 LPM	40 LPM	50 LPM

HFNC Size Guide

HFNC Size	Color	Max Flow
X-Small	BLUE	8 lpm
Small	RED	9 lpm
Medium	ORANGE	10 lpm
Large	PURPLE	23 lpm
X-Large	GREEN	25 lpm
XX-Large	GREY	36 lpm

<u>PSCU (8 South) for the following:</u>

1. All infants < 30 days old or <10kg with respiratory disease
2. **Consider IMC status** for patients who reached max flow for GEN PEDS and RS not improved and/or increasing PEWS score (>4) over 2 assessments

IMC status based on clinical needs

Please use your best clinical judgment, this is meant to serve as a guide
***Use adult protocol for > 18 yo ***
